# Effects of Different Hedgerow Patterns on the Soil Physicochemical Properties, Erodibility, and Fractal Characteristics of Slope Farmland in the Miyun Reservoir Area

**DOI:** 10.3390/plants11192537

**Published:** 2022-09-27

**Authors:** Lei Wang, Jiajun Wu, Jianzhi Xie, Dan Wei, Yan Li, Junqiang Wang, Ting Xu, Zhixin Yang, Liang Jin

**Affiliations:** 1 College of Resources and Environmental Sciences, Agricultural University of Hebei, Baoding 071000, China; 2 Institute of Plant Nutrition, Resources and Environment, Beijing Academy of Agricultural and Forestry Sciences, Beijing 100097, China; 3 Qiqihar Branch of Heilongjiang Academy of Agricultural Sciences, Qiqihar 161000, China

**Keywords:** Miyun reservoir area, hedgerow, soil erodibility, soil physicochemical properties, fractal characteristics

## Abstract

Soil erosion of sloping farmland in the Miyun reservoir area in Beijing has become a serious issue and has threatened the ecological environment and safety of the reservoir area. We used the Taishizhuang Village Non-point Source Pollution Prevention & Control Base in the Miyun reservoir as a study area and performed a comparative analysis of the physicochemical properties of soil of the upper, middle, and lower slopes of the *Scutellaria baicalensis* + *Buchloe dactyloides* plot (Treatment 1, T1), *Morus alba* + *Buchloe dactyloides* plot (Treatment 2, T2), *Salvia miltiorrhiza* + *Cynodon dactylon* plot (Treatment 3, T3), *Platycodon grandiflorus* + *Cynodon dactylon* plot (T4), and a barren land control plot (Control check, CK), to explore how different hedgerow patterns affect the soil’s physicochemical properties, anti-erodibility, and fractal characteristics. We found the following: (1) The primary soil mechanical composition included sand particles in the upper slopes, whereas it was soil fine particles in the middle and lower slopes. (2) The fractal dimension of the slope soil showed a significant negative correlation with sand particles (*R*^2^ = 0.9791) while being positively correlated with silt particles (*R*^2^ = 0.9635) and clay particles (*R*^2^ = 0.9408). (3) All hedgerow patterns increased soil nutrients, with the *Morus alba* + *Buchloe dactyloides* hedgerow plot increasing the soil total nitrogen (STN), soil total phosphorus (STP), and soil organic matter (SOM) content by 213.89–282.69%, 55.56–58.15%, and 29.77–56.04%, respectively. (4) The *Morus alba* + *Buchloe dactyloides* hedgerow plot significantly decreased the soil erodibility factor *K* value, improved soil anti-erodibility, and reduced soil erosion. (5) The *K* value of the soil erodibility was significantly negatively correlated with clay particles, soil fractal dimension, and STP (*p* < 0.01); positively correlated with sand particles; and negatively correlated with silt particles, STN, and SOM. Therefore, the *Morus alba* + *Buchloe dactyloides* hedgerow planting contributes to clay particle conservation, soil nutrient content improvement, soil structure optimization, and soil anti-erodibility enhancement.

## 1. Introduction

The ever-increasing demand for natural resources with the growing economy and population has caused their over-exploitation and has resulted in significant damage to the ecological environment. Land resources play a key role in human survival and development, and human behaviors in the development and utilization process have caused ecological and environmental problems such as soil erosion, soil degradation, and agricultural non-point-source pollution [1,2]. Over 80% of global land degradation is due to soil erosion [3]. The highest soil erosion rate across the world has generally been detected on slopes, especially farmland agricultural slopes [4]. Besides the decrease in soil fertility and productivity, soil erosion on agricultural slopes also causes agricultural non-point-source pollution and creates barriers to soil utilization and agricultural development [5].

The second soil resource investigation in China indicated that the area under soil erosion in the country had reached 3.57 million km^2^, or 37.2% of the national territorial area. The annual soil erosion amount averaged 4.52 billion tons, which accounted for about one-fifth of the global amount, among which 1.415 billion tons was from slope farmland, thereby making up 33% of the total global soil erosion area [6]. China ranks among the countries that are suffering from the most serious soil erosion [7]. Prevention and control of soil erosion not only maintain the harmony between humans and nature, but also are strategies for sustainable development [8,9,10]. As a key water source in Beijing, the Miyun reservoir is pivotal for water security maintenance. However, its upstream and surrounding areas are mainly mountainous regions. Therefore, a large area of slope farmland with a high risk of soil erosion [11] seriously affects the drinking water safety for the residents in Beijing, the capital of China.

The soil erodibility *K* value, a crucial indicator used to measure soil anti-erodibility, is primarily determined by intrinsic soil properties such as particle size composition, moisture, volume weight, dry aggregate structure, and SOM content. Analyzing the internal relation between soil particle size distribution and erodibility helps in determining the mechanism of how soil particle size characteristics affect erosion [12]. Wischmeier specified that a standard plot in the United States is a 22.13 m × 5 m (length × width) leisure plot with a slope of 9%, and he also established a database for American soil erodibility factors based on years of measured data from standard plots across the United States. Being integral to the universal soil loss equation (USLE) and the revised universal soil loss equation (RUSLE), soil mechanical composition, fractal dimension, and nutrient content represent the important indicators for measuring soil erodibility and factors influencing plant growth and development [13].

Hedgerows have been widely used on slope farmlands in China and abroad in social production due to their water and soil conservation effects, such as soil erosion reduction and non-point-source pollution control [14,15,16,17]. Overground and underground hedgerows contribute to 62.25 and 37.75% of runoff reduction along with 60.44 and 39.56% of the sediment reduction advantages on average, respectively [18]. In the black soil region of northeast China, the runoff and sediment reduction benefits brought about by hedgerows were 5.40–10.16% and 51.90–75.72%, respectively [8]. Contrastingly, on the red-soil slope land in southern China, the hedgerows reduced the average runoff and soil erosion by 65.87 and 91.29% under normal rainfall conditions [19]. Studies on slope farmland in the Three Gorges reservoir area showed that hedgerows increased the content of SOM, STN, soil total potassium (STK), and clay particles, thereby enhancing the soil anti-erodibility [20]. Multiple previous studies have demonstrated the significant effect of hedgerows on soil erosion reduction [21,22,23]. However, the effect of hedgerows on soil structure during erosion reduction involves vital questions, such as (1) whether they enhance the soil anti-erodibility by changing the soil structure and (2) whether the different hedgerow patterns impact the effect. Unfortunately, previous studies rarely have answers to these questions. Therefore, taking the Beijing Miyun reservoir as the study area, we measured the soil particle composition and soil nutrients of different hedgerows in this study. This was to explore the spatial distribution characteristics of soil nutrients in different hedgerow patterns, with the soil fractal and erodibility characteristics in four hedgerow patterns being estimated via fractal dimension to provide a reference for enhancing the soil anti-erodibility along with the promotion of soil and water conservation of slopes in the Miyun reservoir.

## 2. Results

### 2.1. Effects of Different Hedgerow Patterns on Soil Mechanical Composition

**Figure 1:**
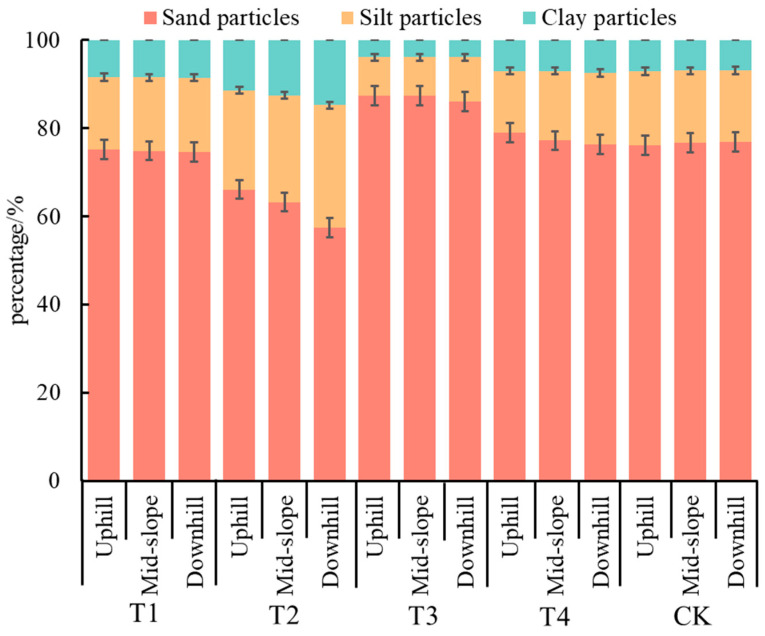
Proportions of sand particles, silt particles, and clay particles under different hedgerow patterns.

The mechanical composition is one of the most important soil physical properties, with the nutrient content and fertilizer retention ability of soil represented by the quantity and mass of soil fine particles (silt particles and clay particles). Therein, clay particles, which constitute part of the soil colloids, are considered as the main carriers of nutrient elements such as N, P, and K due to their large specific surface area and strong ion adsorption force. It can be seen from Figure 1 that the soil particles of slope farmland (CK plot) in the Miyun reservoir area were mainly sand particles (about 76.57%), followed by silt particles (about 16.46%), and clay particles (about 6.97%), whose distribution mainly depended on the nature of soil forming parent materials and environmental factors. Compared to the CK plot, the hedgerow plots T1 and T2 showed a reduction in the percentage of sand particles and an increase in the fine particle content, with the fine particle content in soil under the two hedgerow patterns (T1 and T2) being arranged in the order as T2 > T1. The *Morus alba* + *Buchloe dactyloides* hedgerow pattern could effectively strengthen the anti-erodibility of the topsoil, while also increasing the content of silt and clay particles by 50.86 and 85.21%, respectively, which was accompanied by a significant reduction in silt and clay particles. Compared with the situation in the CK plot, the sand particle content increased while the fine particle content decreased in the hedgerow plots T3 and T4. In the CK plot, the soil hardened due to the loss of fine particles. However, hedgerows could mitigate this and effectively change the soil mechanical composition distribution, thereby preventing soil fine particles from being carried away by runoff.

The spatial distributions of soil mechanical composition in different hedgerow plots were evidently different, where sand particles were mainly distributed on the upper slopes, whereas fine particles were mainly distributed on the middle and lower slopes. In T1, T2, T3, and T4, the fine particle contents in the soil of the upper slopes were 24.88, 33.88, 12.55, and 20.98%, respectively, which were 0.96, 8.55, 0, and 8.6% lower than those on the middle slopes and 2.01, 25.59, 10.60, and 12.77% lower than those on the lower slopes, respectively. Therefore, the fine particle (silt and clay particles) content in the soil of each hedgerow plot showed an increasing trend from the upper slope to the lower slope, but the amplitude of increase varied from plot to plot, which was consistent with previous study results. However, in the CK plot, the fine particle content in soil showed a decreasing trend from the upper slope to the middle and lower slopes, where the fine particle content in the soil on the upper slope was 23.88%, which was 2.27 and 3.6% higher than those on the middle and lower slopes, respectively. This is because in the CK plot, more sand particles were gradually being transferred from the upper slope to the lower slope due to the rainwater erosion without the impeding effect of plants, and consequently, the sand particles gradually increased while the fine particles showed the opposite trend in the upper, middle, and lower slopes.

### 2.2. Effects of Different Hedgerow Patterns on Soil Nutrients

The STN, STP, STK, and SOM are important indexes for measuring soil nutrients. Table 1 displays the descriptive statistical results of the soil nutrients in both the plots with different hedgerow patterns and the CK plot. The differences in each soil nutrient index apparently varied from plot to plot, with the coefficients of variation (CVs) of STN and STP being 2.3–16.2% and 2.0–20.8%, respectively, thereby indicating their weak and moderate degrees of variation. The CVs of STK and SOM were 0.4–3.2% and 0.2–1.2%, respectively, thus indicating their weak degree of variation. From the average value of STN, the content of total nitrogen in the upper slope of the CK plot was the lowest (0.17 g/kg), and that of the lower slope in the T2 plot was the highest (0.75 g/kg), with a difference of 0.58 g/kg. From the average value of STP, the content of total phosphorus in the upper and middle slopes of the CK plot was the lowest (0.75 g/kg), and that in the lower slope of the T2 plot was the highest (1.20 g/kg). The difference between the two was 0.45 g/kg. From the average value of STK, the total potassium content in the upper slope of the CK plot was the lowest (17.12 g/kg), and the content in the lower slope of the T1 plot was the highest (20.24 g/kg). The difference between the two was 3.12 g/kg. From the average value of SOM, the organic matter content in the upper slope of the CK plot was the lowest (8.66 g/kg), and the content in the lower slope of the T2 plot was the highest (17.77 g/kg); the difference between the two was 9.11 g/kg. Additionally, the nutrient contents at different positions under the four different hedgerow patterns were all higher than those in the CK plot.

**Figure 2:**
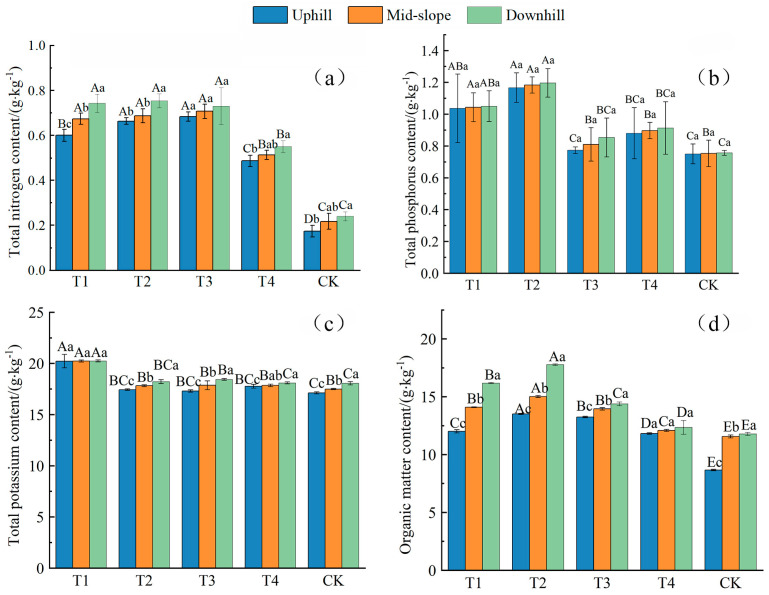
Effects of different hedgerow patterns on soil nutrients. Effects of different hedgerow patterns on total nitrogen content (**a**). Effects of different hedgerow patterns on total phosphorus content (**b**). Effects of different hedgerow patterns on total potassium content (**c**). Effects of different hedgerow patterns on organic matter content (**d**). Note: Different capital letters at the top of the histogram denote the significant differences in soil nutrients in different plots at the same slope position and the same soil layer (*p* < 0.05). Different lowercase letters indicate the significant differences in soil nutrients at different slope positions at the same soil layer in the same plot (*p* < 0.05).

We compared the significant differences in soil nutrients in the four hedgerow plots and the CK plot (*p* < 0.05) to study how the different hedgerow patterns affect the soil nutrient distribution. STN is an important index reflecting the supply status of soil nitrogen. We can see from Figure 2a that at the same slope position in different plots, the STN contents in the four different hedgerow plots were significantly higher than that in the CK plot (*p* < 0.05). The STN contents on the upper, middle, and lower slopes in the T1 plot were 2.46, 2.11, and 2.09 times higher than those in the CK plot, respectively. Furthermore, those in the T2 plot were 2.83, 2.17, and 2.14 times higher than those in the CK plot; those in the T3 plot were 2.94, 2.26, and 2.04 times higher than those in the CK plot; and those in T4 plot were 1.81, 1.37, and 1.29 times higher than those in the CK plot. At different slope positions in the same plot, all five plots were characterized by nitrogen loss on the upper slope and nitrogen enrichment on the middle and lower slopes. In all plots except the T3 plot, the STN content difference between the upper and lower slopes was significant. In the T1 plot, the STN contents on the middle and lower slopes were 12.22 and 23.89% higher than those on the upper slope, respectively. In the T2 plot, the STN contents on the middle and lower slopes were 3.52 and 13.57% higher than those on the upper slope, respectively. In the T3 plot, we found that the STN contents on the middle and lower slopes were 3.41 and 6.83% higher than those on the upper slope, respectively. Furthermore, in the T4 plot, the STN contents on the middle and lower slopes were 5.48 and 13.01% higher than those on the upper slope, respectively. Contrastingly, in the CK plot, the STN contents on the middle and lower slopes were 25 and 38.46% higher than those on the upper slope, respectively. This is mainly because once hedgerows are applied, they reduce the runoffs on the slope surface and increase their STN. Moreover, they impede and intercept runoffs and sediments, thus contributing to the STN enrichment on the middle and lower slopes.

At the same slope position in different plots, the STP content showed similar variation to the STN content (Figure 2b). The STP content in each plot was higher than that in the CK plot, but we found that only the STP contents on the upper, middle, and lower slopes in the T1 and T2 plots were significantly different from those in the CK plot (*p* < 0.05). Moreover, the STP contents on the upper, middle, and lower slopes in T1–T4 plots were 0.03–0.56, 0.08–0.57, and 0.13–0.58 times higher than those in the CK plot; thus, we can sort them as T2 > T1 > T4 >T3 > CK. At different slope positions in the same plot, the STP content underwent losses on the upper slope and enrichment on the middle and lower slopes, with no significant differences being seen.

The STK contents at different slope positions in the T1–T4 plots were all higher than those in the CK plot (Figure 2c). However, it was only in the T1 plot that the STK contents at all slope positions were significantly different from those in the CK plot (*p* < 0.05), with the STK contents on all the slopes being 18.1, 15.64, and 12.11% higher than those in the CK plot, respectively. At different slope positions in the same plot, we can sort the STK content in each plot as upper slope > middle slope > lower slope, with significant differences being seen except in the STK contents on all the slopes in the T1 plot as well as those on the lower and middle slopes in the T4 plot.

The SOM contents at the same slope position in plots with different hedgerow patterns were all significantly higher than those in the CK plot (Figure 2d) (*p* < 0.05), and they could be sorted as T2 > T1 > T3 > T4 > CK. Additionally, the SOM contents at different slope positions in the T2 plot were all significantly higher than those in the other four plots (*p* < 0.05). The SOM contents on the upper, middle, and lower slopes were elevated by 38.68, 21.84, and 37.23%, respectively, under the *Morus alba* + *Buchloe dactyloides* pattern. At different slope positions in the same plot, the SOM content in each plot also underwent losses on the upper slope and enrichment on the lower slope, with significant differences being seen among the SOM contents on all the slopes in all plots except the T4 plot (*p* < 0.05).

### 2.3. Effects of Different Hedgerow Patterns on Various Soil Fractal Characteristics

#### 2.3.1. Effects of Different Hedgerow Patterns on Soil Fractal Dimension

**Figure 3:**
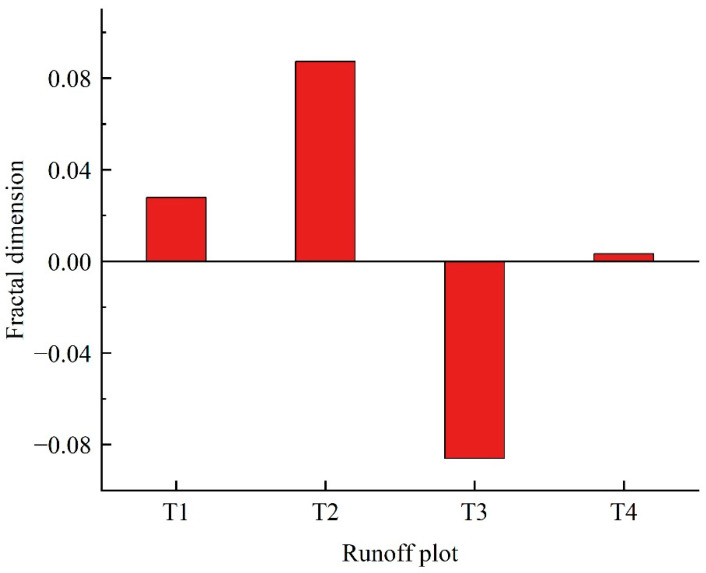
Changes in soil fractal dimensions in different hedgerow plots.

In this study, we analyzed the soil fractal dimensions under different hedgerow patterns and calculated their change features (Figure 3). It could be observed that the variable quantities of the fractal dimension in the T1–T4 plots relative to CK plot were 0.028, 0.087, −0.086, and 0.003, respectively, thereby indicating significant changes in the fractal dimensions in the different hedgerow plots with different hedgerow patterns relative to CK plot. Additionally, we sorted the degrees of the effect of the different hedgerows on the fractal dimension of soil particles in the slope farmland as T2 > T3 > T1 > T4, which was because the controlling effect of hedgerows on soil erosion in the slope farmland is decided by the hedgerow patterns, in which the *Morus alba* + *Buchloe dactyloides* pattern can markedly reduce the soil loss.

#### 2.3.2. Relationship between Fractal Dimension and Soil Particles

**Figure 4:**
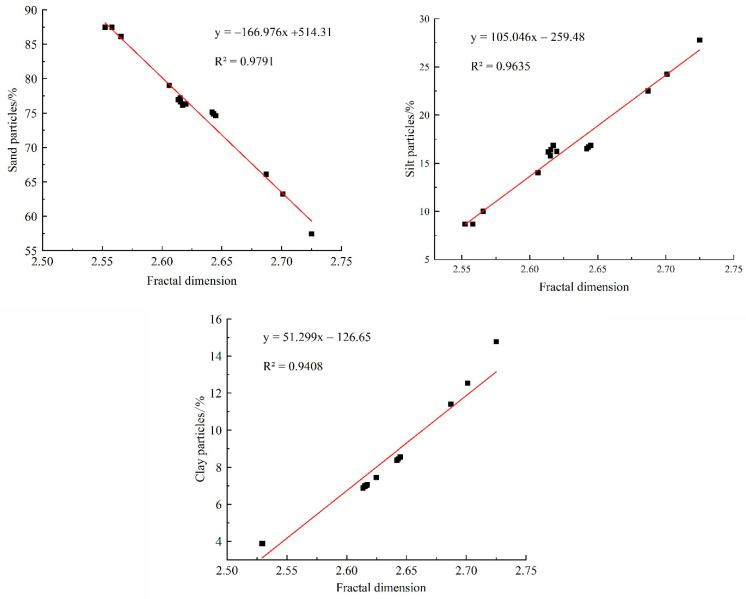
Relationship between soil fractal dimension and particle composition.

The homogeneity of soil texture can be characterized, to some extent, by the fractal dimension of soil particles. Therefore, improving the soil fractal dimension is crucial for reducing soil erosion and controlling the water and soil loss in the slope farmland in the Miyun reservoir area. In this study, we fitted the fractal dimension of soil particles with the content of sand particles, silt particles, and clay particles (Figure 4), where the x-axis represents the changes in the fractal dimension, while the y-axis denotes the changes in the sand particles, silt particles, and clay particles on the slope in each plot. Results showed that the soil fractal dimension showed a significant negative correlation with sand particles (*R*^2^ = 0.9791), while having significantly positive correlations with sand particles (*R*^2^ = 0.9635) and clay particles (*R*^2^ = 0.9408). As shown by the fitting equations, the degrees of the effect of soil particles on the fractal dimension could be sorted as sand particles > silt particles > clay particles, thus indicating that the sand particle content significantly affects the fractal dimension of soil particles, which is also affected by the fine particle content.

### 2.4. Effects of Different Hedgerow Patterns on the Soil Erodibility Factor K

#### 2.4.1. Change Features of Soil Erodibility Factor K

According to the soil erodibility results in different plots, we sorted the soil erodibility *K* values into the different plots as CK > T3 > T4 > T1 > T2, with the CV value of 0.01204–0.74030%, thus indicating a weak degree of variation. The results revealed that T2 was less erodible than the other plots. This is because the differences in root system characteristics under different hedgerow patterns lead to differences in the category and quantity of rhizosphere microorganisms and the soil anti-erodibility. In the *Morus alba* + *Buchloe dactyloides* pattern, *Morus alba* was less erodible due to its ability to intercept rainwater and mitigate its direct impact on the ground. The soil erodibility was differently affected by different hedgerow patterns in a significant manner (*p* < 0.05). As seen in Table 2, the soil erodibility in the T1, T2, and T4 plots was significantly different from that in the CK plot, but the difference between T3 plot and CK plot was insignificant (*p >* 0.05), with the soil erodibility in the T1 and T2 plots significantly differing from those in the T3, T4, and CK plots. More specifically, the soil erodibility was maximum (0.0589 (t·hm^2^·h)/(MJ·mm·hm^2^)) and the minimum (0.0574 (t·hm^2^·h)/(MJ·mm·hm^2^)) in T3 and T2 plots, respectively, with the difference between the maximum and minimum values being the maximum (0.0008 (t·hm^2^·h)/(MJ·mm·hm^2^)) in the T3 plot. We could see that all hedgerow patterns could strengthen the soil anti-erodibility, with the soil anti-erodibility being the strongest in the T2 (*Morus alba* + *Buchloe dactyloides*) plot. This was because hedgerows can effectively intercept surface runoffs and reduce their velocity, and simultaneously, the root systems of *Buchloe dactyloides* significantly affected the soil consolidation via interpenetration, facilitation of soil infiltration, and reduction in slope runoff sediments.

#### 2.4.2. Influencing Factors of Soil Erodibility

**Figure 5:**
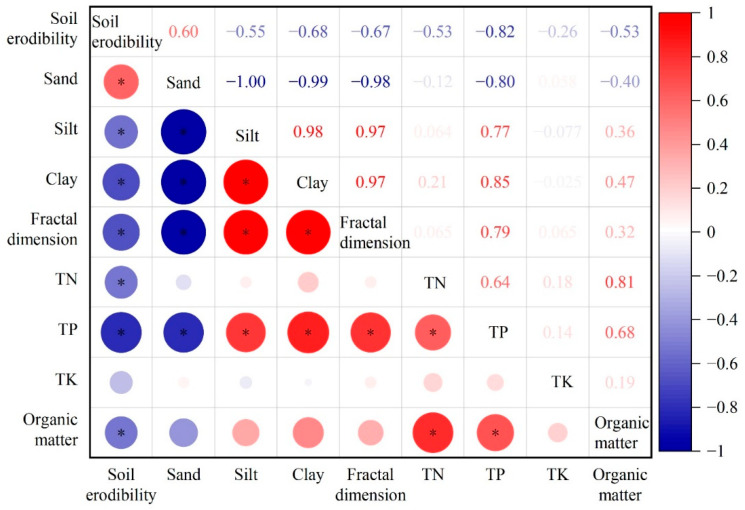
Correlation between soil erodibility and soil physicochemical properties under different hedgerow patterns. * *p* < 0.05.

We performed the correlation analysis of soil erodibility with its influencing factors, e.g., sand particles, silt particles, clay particles, STN, STP, STK, SOM, and fractal dimension (Figure 5). Results showed that the soil erodibility presented highly significant negative correlations with clay particles, soil fractal dimension, and STP (*p* < 0.01); significantly positive correlation with sand particles (*p* < 0.05); and significantly negative correlations with silt particles, STN, and SOM (*p* < 0.05). However, there was no significant correlation with STK (*p* > 0.05). Moreover, the soil fractal dimension showed highly significant positive correlations with silt particles, clay particles, and STP (*p* < 0.01), while exhibiting a significantly negative correlation with sand particles, with the correlation coefficients of 0.971, 0.971, 0.794, and −0.975, respectively. Additionally, the STN showed a highly significant and significant positive correlation with SOM (*p* < 0.01) and STP (*p* < 0.05), with the correlation coefficients being 0.814 and 0.635, respectively. In addition, the STP showed highly significant positive correlations with silt particles, clay particles, soil fractal dimension, and SOM (*p* < 0.01) (correlation coefficients: 0.766, 0.852, 0.794, and 0.677, respectively) and a highly significant negative correlation with sand particles (*p* < 0.01) (correlation coefficient: −0.801).

We subjected each influencing factor to partial correlation analysis with soil erodibility to determine the most fundamental influencing factor and the most effective index of soil erodibility in the Miyun reservoir area. We can see from Table 3 that the soil erodibility was positively correlated with sand particles, STN, and STK, with correlation coefficients of 0.571, 0.200, and 0.213, respectively, while being negatively correlated with silt particles, clay particles, soil fractal dimension, STP, and SOM, with correlation coefficients of −0.543, −0.619, −0.656, −0.564, and −0.036, respectively. Given that the loss of soil nutrients mainly results from particle migration and runoff washing, the content of soil clay particles—one of the important carriers of soil nutrients—significantly affects the soil anti-erodibility. Hence, how hedgerows affect the soil erodibility factor *K* and the fractal characteristics of soil particles with the increase in their plantation period need further exploration.

## 3. Materials and Methods

### 3.1. Overview of the Study Area

**Figure 6:**
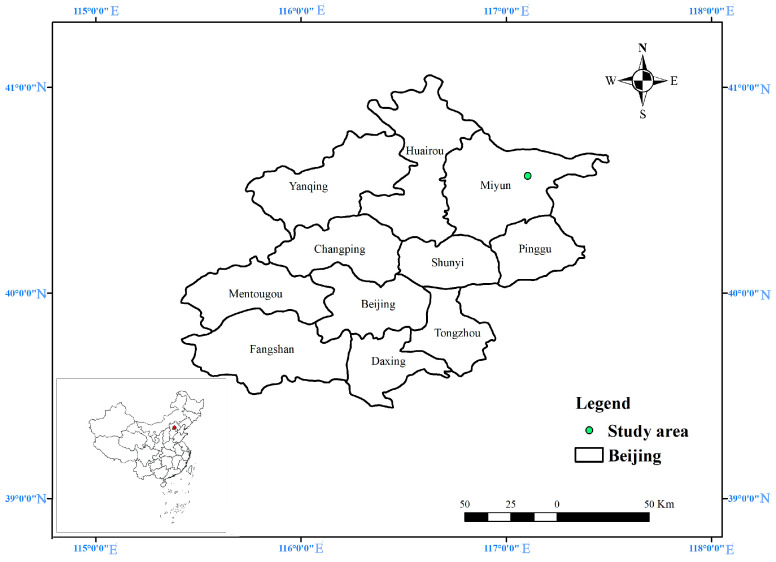
Location map of runoff plots.

The test site was located in Taishizhuang Village Non-point Source Pollution Prevention & Control Base (117°6′42.08″ E, 40°32′22.02″ N) around Miyun reservoir, Miyun District, Beijing, China (Figure 6). The river in the north was the Chao River, and the river in the south was the Qingshui River. The climate type belonged to the warm temperate semi-humid monsoon climate. The average annual temperature of multiple years was 9–10.5 °C. The frost-free period was 180 days. The average annual rainfall of multiple years was about 624 mm and was mostly concentrated from June to September. The rainfall in the three months of the flood season accounted for ~65–75% of the annual rainfall. In the study area, sandy loam soil was the dominant soil type, with its basic physicochemical properties being: pH = 6.33, SOM content = 9.97 g/kg, STN content = 0.448 g/kg, rapidly available phosphorous content = 4.55 mg/kg, and rapidly available potassium content = 45.9 mg/kg [24].

### 3.2. Experimental Design

Based on the principles of the adaptability of plants to local land and climatic conditions, six ecological plants, perennial herbaceous or ligneous plants, were chosen in the inter-cropping experiment, namely *Morus alba L*., *Salvia miltiorrhiza Bunge*, *Buchloe dactyloides (Nutt.) Engelm*., *Scutellaria baicalensis Georgi*, *Platycodon grandiflorus (Jacq.) A. DC*., and *Cynodon dactylon (Linn.) Pers*. The plants were characterized by their good water and soil conservation effects, consistency between the prosperous growing season and concentrated rainy season, and high economic values. Moreover, these were chosen due to their economic and landscape values and also since they did not need fertilization during their growth period. *Morus alba* was planted via transplantation, while *Salvia miltiorrhiza*, *Buchloe dactyloides*, *Scutellaria baicalensis*, *Platycodon grandiflorus*, and *Cynodon dactylon* were planted via direct seeding.

**Figure 7:**
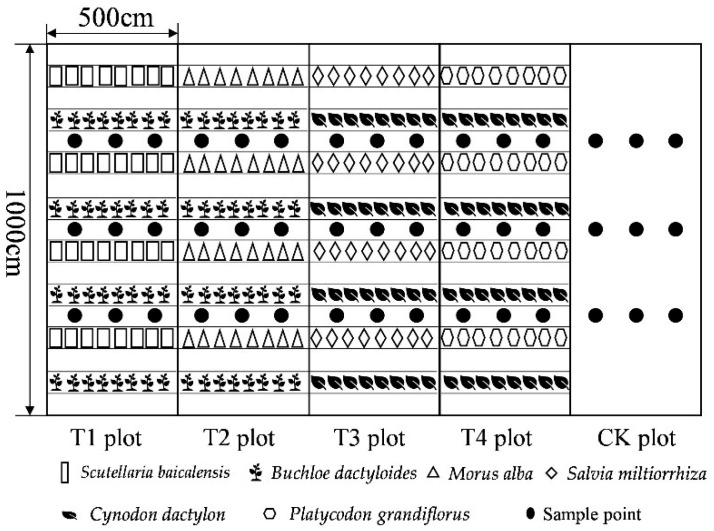
Schematic diagram of sampling points in runoff plots.

A total of five runoff plots were selected for this experiment, i.e., Scutellaria baicalensis + Buchloe dactyloides plot (T1), Morus alba + Buchloe dactyloides plot (T2), Salvia miltiorrhiza + Cynodon dactylon plot (T3), Platycodon grandiflorus + Cynodon dactylon plot (T4), and barren land control (CK) plot, with a slope of 10° and a horizontally projected area of 50 m^2^ (length: 10 m, width: 5 m). There were five treatments and three replicates for each treatment. In April 2019, all six plants were arranged in runoff plots with a plant and hedgerow spacing of 15 cm and 100 cm, respectively. In each runoff plot, each plant species was planted in a 1:1 layout. Eight rows were arranged on the upper, middle, and lower slopes, and the runoff plots and sampling points are displayed in Figure 7. Weeds on the bare land (CK) were regularly cleared by professionals to avoid errors. The hedgerow composite patterns and the hedgerow growth status in each plot of 2021 are listed in Table 4.

### 3.3. Experimental Method

#### Sample Collection and Treatment

In the last ten days of September 2021, samples were collected without weeding measures, provided that the sky cleared up for over three days after raining. In this process, the typical sampling method was implemented at the appropriate soil moisture when the soil did not adhere to the shovel or suffer deformation on contact. Each sampling point was 125 cm from the side wall and 0–20 cm from the hedgerow, and this was to reduce errors. Moreover, each runoff plot was divided into three slope positions: upper, middle, and lower slopes. Three sampling points were arranged from each, with an equal spacing from the left to the right (125 cm). After the surface covers were removed, soil samples at the depth of 0–20 cm were collected using a soil drill, then placed into bags, and finally numbered.

The soil samples from experimental plots were carried back to the laboratory, air dried, and sieved for later use. After being air-dried, the plant roots were picked out. The soil mechanical composition was determined via a laser particle size analyzer (CLY-2000, Dandong Chaowei Silt Technology Co., Ltd., Dandong, China). Additionally, soil particles were divided into three grades, namely sand particles (2–0.05 mm), silt particles (0.05–0.002 mm), and clay particles (<0.002 mm), based on the American soil composition grading standards. Soil nutrients were detected through conventional soil agrochemical analysis methods: The SOM, STN, STP, and STK content were determined via the potassium dichromate volumetry–outside heating method, sulfuric acid–catalyst digestion method, sodium hydroxide melting–Mo-Sb colorimetry, and sodium hydroxide melting–flame photometry [25], respectively.

### 3.4. Calculation of Soil Fractal Dimension

In this study, the soil fractal dimension was solved through the volume fractal dimension formula proposed by Liu [26], which is as follows:

(1) $$\frac{V\left(r<R\right)}{VT}={\left(\frac{R}{\lambda V}\right)}^{3-D}$$

Logarithms were taken from both sides of the above formula to obtain

(2) $$\mathrm{lg}\left[\frac{V\left(r<R\right)}{VT}\right]=\left(3-D\right)\mathrm{lg}\left(\frac{R}{\lambda V}\right)$$

where *r*, *R*, *V* (*r* < *R*), *VT*, and *λV* indicate the soil particle size, the arithmetic average of the upper and lower limit values of one particle size grade, the cumulative volume of <*R* soil particles, the total volume of soil particles, and the upper limit value (equal to the maximum particle size) of all particle size grades, respectively. First, lg[*V*(*r* < *R*)/*VT*] was calculated as the y-coordinate and lg(*R*/*λV*) was calculated as the x-coordinate, and they were then linearly fitted to obtain the slope as 3-*D*, thus solving the *D* value.

### 3.5. Calculation of Soil Erodibility Factor K

The soil erodibility *K* value was calculated as per the formula proposed by Williams [27] in the EPIC model as follows:

(3) $$K=\left(0.2+0.3e^{-0.0256SAN\left(1-\frac{SIL}{100}\right)}\right){\left(\frac{SIL}{CLA+SIL}\right)}^{0.3}\left(1-\frac{0.25C}{C+e^{3.72-2.95C}}\right)\left(1-\frac{0.7SN}{SN+e^{-5.51+22.9SN}}\right)$$

where *SAN*, *SIL*, *CLA*, and *C* represent the sand particle content, the silt particle content, the clay particle content, and the SOM content, respectively (unit: %). *SN* = 1 − *SAN*/100, and the calculation result should be converted into an SI unit, i.e., (T·hm^2^·h)/(MJ·mm·hm^2^).

### 3.6. Statistical Analysis

The data were recorded and processed using Excel, and comparisons of the groups were performed by a one-way analysis of variance (one-way ANOVA) with a Duncan multiple variable test (*p < 0.05*). The correlation coefficients were calculated by the SPSS 26 software (SPSS Inc., Chicago, IL, USA) through Pearson’s correlation and a two-tailed t test (*p* < 0.05 and *p* < 0.01). Origin 2021 was used for plotting data.

## 4. Discussion

Soil mechanical composition serves as the basis for soil structure, with the content of soil particles being closely associated with soil nutrients, soil fractal dimension, and soil erodibility. According to the existing studies, hedgerows can change the soil mechanical composition by intercepting soil particles, but their intercepting effects vary with soil particles of different size grades [28]. Yang discovered that, as compared to the bare control land, hedgerows can reduce the sand particle content and elevate the content of silt particles and clay particles [29]. Li investigated two types of hedgerows, *Gardenia jasminoides* and *Lolium perenne L*., in the typical lime soil slope farmland in the Xiangxi River drainage basin of the Three Gorges reservoir area and found that the hedgerow system can effectively increase the content of fine particles in the soil plow layer [30]. In this study, the experimental results of T1 (*Scutellaria baicalensis* + *Buchloe dactyloides*) and T2 (*Morus alba* + *Buchloe dactyloides*) plots were consistent with previous studies, which showed that fine particles increased while sand particles decreased in soil. Contrastingly, it was completely opposite in the T3 (*Salvia miltiorrhiza* + *Cynodon dactylon*) and T4 (*Platycodon grandiflorus* + *Cynodon dactylon*) plots. This was because the plant coverage was large due to the developed root systems in the topsoil of the *Cynodon dactylon* slope surface, which led to soil loosening, porosity increment, and a reduction in the blocking and controlling effect on fine particles.

Soil nutrients, the basis for land productivity, are important indexes for evaluating soil quality [31]. In a study on purple soil, Wang discovered that the SOM contents in front of hedgerows in the same plot were sorted as lower slope > middle slope > upper slope [32]. Yan (2017) investigated the effects of soil nutrients in the slope farmland in the western Liaoning Province and found that the STN and STP content could be increased by all hedgerows, among which *Medicago sativa L.* hedgerows had the best effect. When studying the slope farmland in the Three Gorges reservoir area [33], Lei concluded that the STN, acid-hydrolyzable nitrogen (AHN), and soil organic carbon (SOC) content all can be significantly increased by the two-belt *Morus alba* (TM) contour hedgerow, two-belt composite *Morus alba* vetiver (TCMV) hedgerow, two-belt composite *Morus alba* flower (TCMA) hedgerow, seven-year two-belt *Morus alba* (7YTM) contour hedgerow, and seven-year boundary *Morus alba* (7YBM) hedgerow (*p* < 0.05) [34]. Our results were consistent with these earlier mentioned studies, and we observed that all four hedgerow patterns can increase the soil nutrient content to different degrees, which, however, differs from the results of Zheng. They carried out a hedgerow-crop intercropping experiment during 2012–2018 and discovered that the TN, TP, and SOM contents recorded in the hedgerow plots showed unclear variation trends relative to the control plot, by being slightly high or low at one time or another, thereby having little effect on the total nutrient content in the topsoil [14]. This was mainly due to the differences in environment, cultivation management, and hedge species and the lack of competition of crops for soil nutrients in this experiment. Among the four different hedgerow patterns, we achieved a favorable comprehensive blocking and controlling effect on soil nutrients in the T2 (*Morus alba* + *Buchloe dactyloides*) plot, which was consistent with the results of Lu [35], who showed that a hedgerow consisting of shrubs and herbaceous plants produces a relatively good blocking and controlling effect on soil nutrients.

Li explored the soil fractal dimension of a hedgerow system in the Three Gorges reservoir area and discovered that it showed a highly significant positive correlation with the content of the clay and silt particles in the soil (*p* < 0.01) and a significantly negative correlation with the sand particle content in the soil [36]. Huang found that the soil fractal dimension showed significant linear correlations with the variable content of fine clay particles, coarse clay particles, and coarse sand particles, with the soil fractal dimension in the hedgerow plots being higher than that in the bare control plot [37]. Li concluded that in the Three Gorges reservoir area, the soil fractal dimension showed highly significant positive correlations with the concentration of clay particles (*R*^2^ = 0.93) and silt particles (*R*^2^ = 0.74) in the soil (*p* < 0.01) and a highly significant negative correlation with the content of sand particles in soil (*R*^2^ = 0.78) [38]. We also shared the same conclusion in this experiment, where the soil fractal dimension showed a significantly negative correlation with the sand particles while showing a significantly positive correlation with both sand and clay particles.

Soil erodibility is an important indicator for measuring the sensitivity of soil erosion and also an important parameter for determining the difficulty of soil transportation and dispersion under exogenic forces. The smaller the *K* value, the stronger the soil erosion resistance [39,40]. In a study on the typical metamorphic soil in the Loess Plateau, Yang discovered that the soil erodibility parameter of the hedgerow slope (3.58 g∙N^−1^∙m^−1^) was greater than that of the control slope (2.83 g∙N^−1^∙m^−1^), thereby reflecting that soil erodibility can be enhanced by planting hedgerows [41]. Chen explored the effect of *Andrographis paniculata* (Burm. f.) Nees hedgerows on the soil erodibility and found that the soil erodibility was reduced by planting hedgerows, with the soil erodibility factor *K* showing a significantly positive correlation with the sand particle content and a significantly negative correlation with the content of sand particles, clay particles, and SOC as well as the fractal dimension of the particles [42]. Consistent with previous study results, all four hedgerow patterns in this study can enhance the soil anti-erodibility to different degrees. Furthermore, the soil erodibility was positively correlated with the content of sand particles, STN, and STK but negatively correlated with the content of silt particles, clay particles, STP, and SOM, as well as the soil fractal dimension. In addition, due to the dynamic changes in soil physicochemical properties, soil erodibility also changed [43]. In addition, the soil erodibility factor *K* and soil fractal dimension were limited by various factors, including the soil mechanical composition, soil nutrients, and tillage.

Soil physicochemical properties are prone to annual and interannual alterations due to changes in the climate and farming system. Moreover, there is no exception for the fractal characteristics and erodibility of soil particles, which, meanwhile, are co-restricted by many factors. The influencing mechanism of hedgerows on the agricultural non-point-source pollution of slope farmlands was quite complicated and affected by numerous factors, including the species of crops planted, the soil humus content, the aggregate content, and the artificial tillage method. With the increase in the plantation period of hedgerows, a long-term study must be carried out regarding the multi-factor combined effects on soil erosion.

## 5. Conclusions

From the effects of different hedgerow patterns on soil physicochemical properties, erodibility, and fractal characteristics, we found that the soil mechanical composition and nutrient contents in the same plot were apparently different, with sand particles playing a dominant role in the soil mechanical composition. Sand particles were distributed on the upper slope, while fine particles were mainly distributed on the middle and lower slopes in each plot. Moreover, the soil nutrient content in each plot could be sorted as lower slope > middle slope > upper slope. In the CK plot, the difference between the SOM content on the upper slope and that on the lower slope was the maximum, where the latter was 38.68% higher than the former. The T1, T2, and T4 can increase the soil fractal dimension, and the soil fractal dimension increase amplitude was the maximum (0.087) in the T2 plot. The soil fractal dimension on the slope showed a significantly negative correlation with sand particles (*R*^2^ = 0.9791) and significantly positive correlations with the silt (*R*^2^ = 0.9635) and clay particles (*R*^2^ = 0.9408). Additionally, the soil nutrients can be increased by different hedgerow patterns, among which the *Morus alba* + *Buchloe dactyloides* hedgerow pattern increased the contents of STN, STP, and SOM by 213.89–282.69%, 55.56–58.15%, and 29.77–56.04%, respectively. The *Morus alba* + *Buchloe dactyloides* hedgerow pattern can (1) markedly reduce the soil erodibility *K* value, (2) strengthen the soil anti-erodibility, and (3) reduce water and soil loss in the experimental plot. We could sort the degrees of the effect of different hedgerow patterns on the *K* value as T3 > T4 > T1 > T2. Furthermore, the soil erodibility *K* value showed highly significant negative correlations with the content of clay particles and STP, and the soil fractal dimension (*p* < 0.01) exhibited a significantly positive correlation with the content of sand particles and significantly negative correlations with the content of silt particles, STN, and SOM. *Morus alba* is an economical fruit tree species exclusively grown in China. Our study results revealed that in the slope farmland in the Miyun reservoir area, the *Morus alba* + *Buchloe dactyloides* hedgerow pattern can (1) effectively control the loss of clay particles, (2) increase the soil nutrient content, (3) optimize the soil structure, and (4) strengthen the soil anti-erodibility, along with positive effects, including the increase in local farmers’ income and improvement of the local ecological environment.

## Figures and Tables

**Table 1:** Descriptive statistics of the STN, STP, STK, and SOM contents under different hedgerow patterns.

		STN		STP		STK		SOM	
Plot	Slope Position	Value (g·kg^−1^)	CV (%)	Value (g·kg^−1^)	CV (%)	Value (g·kg^−1^)	CV (%)	Value (g·kg^−1^)	CV (%)
T1	Upper	0.60 ± 0.026	4.4%	1.04 ± 0.216	20.8%	20.22 ± 0.654	3.2%	12.01 ± 0.139	1.2%
	Middle	0.67 ± 0.025	3.7%	1.04 ± 0.091	8.7%	20.23 ± 0.095	0.5%	14.09 ± 0.032	0.2%
	Lower	0.74 ± 0.040	5.4%	1.05 ± 0.096	9.2%	20.24 ± 0.096	0.5%	16.18 ± 0.046	0.3%
T2	Upper	0.66 ± 0.015	2.3%	1.17 ± 0.093	8.0%	17.43 ± 0.078	0.5%	13.51 ± 0.055	0.4%
	Middle	0.69 ± 0.031	4.5%	1.18 ± 0.051	4.3%	17.82 ± 0.093	0.5%	15.01 ± 0.087	0.6%
	Lower	0.75 ± 0.031	4.1%	1.20 ± 0.089	7.5%	18.22 ± 0.191	1.1%	17.77 ± 0.071	0.4%
T3	Upper	0.68 ± 0.021	3.1%	0.77 ± 0.021	2.7%	17.30 ± 0.110	0.6%	13.24 ± 0.061	0.5%
	Middle	0.71 ± 0.32	4.6%	0.81 ± 0.105	13.0%	17.86 ± 0.427	2.4%	13.95 ± 0.107	0.8%
	Lower	0.73 ± 0.082	11.2%	0.85 ± 0.122	14.3%	18.43 ± 0.081	0.4%	14.39 ± 0.164	1.1%
T4	Upper	0.49 ± 0.025	5.2%	0.88 ± 0.161	18.3%	17.77 ± 0.190	1.1%	11.82 ± 0.065	0.6%
	Middle	0.51 ± 0.021	4.1%	0.90 ± 0.051	5.7%	17.85 ± 0.121	0.7%	12.09 ± 0.089	0.7%
	Lower	0.55 ± 0.026	4.8%	0.91 ± 0.165	18.1%	18.09 ± 0.098	0.5%	12.35 ± 0.595	4.8%
CK	Upper	0.17 ± 0.025	14.5%	0.75 ± 0.062	8.3%	17.12 ± 0.097	0.6%	8.66 ± 0.062	0.7%
	Middle	0.22 ± 0.035	16.2%	0.75 ± 0.083	11.1%	17.49 ± 0.061	0.4%	11.56 ± 0.131	1.1%
	Lower	0.24 ± 0.020	8.3%	0.76 ± 0.015	2.0%	18.06 ± 0.162	0.9%	11.79 ± 0.131	1.1%

Note: Value = mean ± standard error (SE), CV = σ/μ, σ represents standard error, μ represents mean.

**Table 2:** Variable parameters of soil erodibility characteristics under different hedgerow patterns.

Plot	Mean Value (t·hm^2^·h·MJ^−1^·mm^−1^·hm^−2^)	Minimum Value (t·hm^2^·h·MJ^−1^·mm^−1^·hm^−2^)	Maximum Value (t·hm^2^·h·MJ^−1^·mm^−1^·hm^−2^)	CV (%)
T1	0.0576c	0.0575	0.0576	0.0120%
T2	0.0575c	0.0574	0.0576	0.1846%
T3	0.0584ab	0.0581	0.0589	0.7403%
T4	0.0580bc	0.0576	0.0583	0.5899%
CK	0.0585a	0.0585	0.0586	0.0830%

Note: Different letters represent significant difference among different plots (*p* < 0.05).

**Table 3:** Partial correlation between soil erodibility and soil physicochemical properties under different hedgerow patterns.

	Sand Particle	Silt Particle	Clay Particle	Fractal Dimension	STN	STP	STK	SOM
Correlation	0.571	−0.543	−0.619	−0.656	0.200	−0.564	0.213	−0.036
Significance (two-tail)	0.033	0.045	0.018	0.011	0.494	0.036	0.464	0.904

**Table 4:** Hedgerow patterns in different runoff plots.

Plot	Hedgerow Pattern	Plant Height (cm)	Slope	Land Use Type
T1	*Scutellaria baicalensis* + *Buchloe dactyloides*	40	10	Forest and grass land
T2	*Morus alba* + *Buchloe dactyloides*	230	10	Forest and grass land
T3	*Salvia miltiorrhiza* + *Cynodon dactylon*	45	10	Forest and grass land
T4	*Platycodon grandiflorus* + *Cynodon dactylon*	50	10	Forest and grass land
CK	Bare land	-	10	Bare land

## Data Availability

Not applicable.
